# Medical College Notes

**Published:** 1894-08

**Authors:** 


### Medical College Notes.

---

The Birmingham Medical College, incorporated under the laws of Alabama, June 9, 1894, has recently sent out its first annual announcement. It is governed by a board of regents and has a well-equipped faculty, among which we notice that Dr. John D. S. Davis is professor of the principles and practice of surgery and clinical surgery ; Dr. W. E. B. Davis is professor of gynecology and abdominal surgery, and Dr. R. N. Cunningham is professor of physical diagnosis and clinical medicine. Birmingham is the ideal place for medical teaching in Alabama, and we have no doubt that this new college will prove a success.

---

The University of Pennsylvania will soon add three new buildings to its plant, all to be connected with the medical department, thus increasing its efficiency. These are the Agnew memorial wing to the hospital, the William Pepper clinical laboratory and a main ward to the maternity hospital, that will connect with the smaller wings already constructed. The clinical laboratory is endowed by Dr. William Pepper as a memorial to his father. It is to be used for post-graduate study and original scientific research. It will be four stories high with a basement. In the latter will be placed the heating and ventilating apparatus. The first, second and third stories will be fitted up as laboratories, and the fourth will contain rooms for the curator and janitor.

The Agnew memorial will be erected by the widow of Dr. D. Hayes Agnew, and will be equipped as a complete surgical department, without regard to expense.

The addition to the maternity hospital will contain a basement, kitchen and dining-room, while wards and baths occupy the three stories above, for the accommodation of patients previous to their treatment in the present wards.

The University of Pennsylvania will soon boast of the most complete medical department in America, if not in the world.

---

In the Kansas City Medical College Dr. David R. Porter has resigned as professor of principles and practice of medicine and clinical medicine, and Dr. Joseph Sharp has been chosen to succeed him. Dr. J. D. Griffith has been appointed dean, vice Dr. D. R. Porter, resigned. Dr. Frank W. Rathbone has been chosen to fill Dr. Sharp's former chair, that of professor of therapeutics and clinical medicine. Dr. George Mosher has been made professor of obstetrics, and Dr. Herman Pearse, who is Dr. Lanphear's successor as editor of the *Medical Index*, has been advanced to the professorship of anatomy. Dr. Andrew Fulton takes the chair of operative and clinical surgery. Dr. T. J. Beattie is appointed lecturer on diseases of women and children, and Dr. A. H. Cordier is advanced to the lectureship on abdominal surgery.

---

The medical department of the University of Wooster, at Cleveland, O., has a new hospital and dispensary almost completed. New laboratories also have been equipped, and that for bacteriology was ordered from Europe alone costing over $3,000. The faculty also has been greatly augmented in numbers, as will appear by consulting the card on page xxiii. of the Journal.

